# CIRSE Standards of Practice on Thermal Ablation of Bone Tumours

**DOI:** 10.1007/s00270-022-03126-x

**Published:** 2022-03-29

**Authors:** Anthony Ryan, Caoimhe Byrne, Claudio Pusceddu, Xavier Buy, Georgia Tsoumakidou, Dimitrios Filippiadis

**Affiliations:** 1 Division of Interventional Radiology, Department of Radiology, University Hospital Waterford, Waterford City, Ireland; 2 Division of Interventional Radiology, Department of Oncologic Radiology, Businco Hospital, Cagliari, Italy; 3 Department of Diagnostic and Interventional Radiology, Institut Bergonié, Regional Comprehensive Cancer Centre, 33076 Bordeaux, France; 4 Department of Diagnostic and Interventional Radiology, CHUV, Lausanne, Switzerland; 5 2nd Department of Radiology, University General Hospital “ATTIKON”, Athens, Greece

**Keywords:** Interventional pain management, Radiofrequency ablation, Cryoablation, Microwave ablation, HIFU, Laser ablation, Bone tumours

## Abstract

**Background:**

Percutaneous thermal ablation is an effective, minimally invasive means of treating a variety of focal benign and malignant osseous lesions. To determine the role of ablation in individual cases, multidisciplinary team (MDT) discussion is required to assess the suitability and feasibility of a thermal ablative approach, to select the most appropriate technique and to set the goals of treatment i.e. curative or palliative.

**Purpose:**

This document will presume the indication for treatment is clear and approved by the MDT and will define the standards required for the performance of each modality. CIRSE Standards of Practice documents are not intended to impose a standard of clinical patient care, but recommend a reasonable approach to, and best practices for, the performance of thermal ablation of bone tumours.

**Methods:**

The writing group was established by the CIRSE Standards of Practice Committee and consisted of five clinicians with internationally recognised expertise in thermal ablation of bone tumours. The writing group reviewed the existing literature on thermal ablation of bone tumours, performing a pragmatic evidence search using PubMed to search for publications in English and relating to human subjects from 2009 to 2019. Selected studies published in 2020 and 2021 during the course of writing these standards were subsequently included. The final recommendations were formulated through consensus.

**Results:**

Recommendations were produced for the performance of thermal ablation of bone tumours taking into account the biologic behaviour of the tumour and the therapeutic intent of the procedure. Recommendations are provided based on lesion characteristics and thermal modality, for the use of tissue monitoring and protection, and for the appropriately timed application of adjunctive procedures such as osseus consolidation and transarterial embolisation.

**Results:**

Percutaneous thermal ablation has an established role in the successful management of bone lesions, with both curative and palliative intent. This Standards of Practice document provides up-to-date recommendations for the safe performance of thermal ablation of bone tumours.

## Introduction

The CIRSE Standards of Practice Committee established a writing group which was tasked with producing up-to-date recommendations for the performance of thermal ablation of bone tumours. It is neither a clinical practice guideline nor a systematic review of the literature. CIRSE Standards of Practice documents are not intended to impose a standard of clinical patient care, but recommend a reasonable approach to, and best practices for, the performance of thermal ablation of bone tumours. Institutions should regularly review their internal procedures for development and improvement, taking into account international guidance, local resources and regular internal morbidity and mortality reviews. A summary of key recommendations on thermal ablation of bone tumours can be found in Appendix 1.

## Methods

The writing group, which was established by the CIRSE Standards of Practice Committee, consisted of 5 clinicians with internationally recognised expertise in thermal ablation of bone tumours. The writing group reviewed the existing literature on thermal ablation of bone tumours, performing a pragmatic evidence search using PubMed to search for publications in English and relating to human subjects from 2009 to 2019. Selected studies published in 2020 and 2021 during the course of writing these standards were subsequently included. The final recommendations were formulated through consensus.

## Background

Percutaneous thermal ablation is an effective, minimally invasive means of treating a variety of focal benign and malignant osseous lesions [1–5] and should be included in the treatment algorithm alongside surgery, systemic therapies and radiotherapy. The latter have shortcomings, particularly in the management of osseus metastases. *Radiation therapy (RT)* has been considered the standard treatment for pain relief and local control of bone metastases; however, it is limited by a delayed onset of pain relief, the number of adequately radiosensitive tumour types and an upper dose limit, the latter especially important in residual or recurrent tumours [6]. Post-radiation fracture may occur, the majority in the first 4 months post-treatment, especially after stereotactic beam radiation therapy (SBRT) or stereotactic radiosurgery of lytic lesions [7, 8]. RT may be complicated by osteonecrosis or neural injury [9]. RT may be contraindicated owing to limited cumulative tolerance of surrounding radiosensitive organs, such as bowel or spinal cord. Unlike RT, ablative procedures are effective in reducing pain within 48–72 h of treatment [5, 10] and can be repeated in cases of residual or recurrent disease. *Surgical osseous metastasectomy* is technically challenging, associated with high complication and morbidity–mortality rates and requires prolonged recovery, delaying the use of systemic therapies [1]. Surgery is excessively invasive and morbid for small focal lesions such as osteoid osteoma (OO) and for palliating painful osseous metastases.

To determine the role of ablation in individual cases, each patient’s overall status and target lesion(s) should be discussed in a multidisciplinary team (MDT) setting involving the appropriate subspecialty physicians, specifically to assess the suitability and feasibility of a thermal ablative approach, to select the most appropriate technique and to set the goals of treatment, i.e. curative in benign and certain limited malignant settings (including oligometastatic, oligorecurrent or oligoprogressive disease) and palliative in the remaining malignant settings (including the prevention of skeletal-related events [11]). This discussion is especially important in oligometastatic disease and in spinal lesions where multiple treatment modalities may be required to achieve pain relief and stabilisation.

This document will presume the indication for treatment is clear and approved by the MDT and will define the standards required for the performance of each modality. The advantages of each modality in particular settings will be discussed. Standards for the use of tissue monitoring and protection in preventing non-target destruction of adjacent sensitive structures will be emphasised, as will the optimal timings with respect to other interventions, e.g. embolisation in hypervascular lesions or osseous consolidation [2, 3].

### Indications Based on Biologic Behaviour and Therapeutic Intent

Treatment with *Curative Intent*:

- For benign tumours: Successfully treated lesions include OO, osteoblastoma, eosinophilic granuloma, chondroblastoma and aneurysmal bone cyst [12–20].
- For primary malignant bone tumours in carefully selected cases of small (< 3 cm), slow-growing lesions in non-surgical candidates or in patients refusing surgery.
- In selected patients presenting with oligometastatic, oligorecurrent and oligoprogressive disease, i.e. < 3–5 potentially treatable metastases, each < 3 cm [21], including ablation specifically directed at preventing compromise of adjacent critical structures due to tumour progression, particularly in spinal lesions [22].

Ablation is offered with palliative intent to treat painful metastases refractory to, or unsuitable for, pharmacologic management, RT or surgery [3, 6, 23–25].

### Brief Overview of Each Modality’s Mode of Action

All the heat-based technologies effect cell death via the common end-points of protein denaturation and coagulative necrosis.

*Radiofrequency ablation (RFA)* uses an applicator to deliver high frequency alternating current (400 and 500 kHz) to the target tissue causing ionic agitation and frictional heat (to temperatures of 60–100 °C). The volume of the ablation zone achieved is dependent on target tissue impedance, and adjacent perfusion and ventilation.

*Microwave ablation (MWA)* uses an electrical current produced by a 915-MHz or 2.45-GHz generator and delivered via a water-cooled interstitial antenna to produce a local non-ionising electromagnetic field which interacts with dipolar molecules causing frictional heating.

*LASER photocoagulation or laser interstitial thermotherapy (LITT)* uses infrared energy transmitted through a bare-tip 400–600 µm optical fibre to ablate tumoural tissue via photocoagulation secondary to heat scatter.

*High-intensity focused ultrasound (HIFU)* uses high-energy ultrasound (US) to ablate tissue. The beam (200 kHz–4 MHz) is focused on the target tissue within which beam attenuation causes heating to 65–85 °C [26].

*Cryoablation (CA)* uses extreme cold to destroy tumours. Delivered under high pressure via a cryoprobe, rapid expansion of argon gas produces a sudden profound temperature drop (below − 183 °C) (the Joule–Thompson phenomenon), causing intra- and extracellular water to freeze, disrupting cell membranes and organelle structure. During subsequent passive thawing, fluid shift occurs from the interstitium into tumour cells, causing cell rupture, and rendering further water available for freezing during the next freeze phase. Cellular necrosis is systematically achieved with temperatures below − 40 °C. Intravascular ice crystals, direct endothelial freezing, microthrombi and post-ablation oedema cause indirect ischaemia. Tumour cells, fibroblasts and collagen have a greater resistance to a single freezing exposure than healthy osteocytes [27–29], hence the need for freeze–thaw–freeze cycles.

*Regardless of modality*, reduction in tumour bulk, decrease in local inflammatory mediators and induction of osteoclast activity with resultant sclerosis all play a role in pain palliation [30], as does inclusion of nerve-rich periosteum in the ablation zone. Specifically in the case of OO, ablation of periosteum with the nidus results in interrupted production of inflammatory prostaglandins and prostacyclins.

### Recommendations Based on Lesion Characteristics and Thermal Modality

- *RFA* is indicated for osteolytic or mixed osteolytic–osteoblastic lesions with no, or a small, extra-osseous component [31]. Where an extra-osseus soft tissue component exists, ablation of the soft tissue–bone interface can achieve pain palliation.
- *MWA* reaches higher intra-tumoural temperatures than other modalities and is less affected by tissue conductivity/impedance variables and perfusion-mediated tissue cooling. Thus, sclerotic lesions are better treated with MWA instead of RFA, as the latter is rendered relatively ineffective by the high impedance of sclerotic bone [32]. Further advantages of MWA include larger tumour ablation volumes achievable in shorter times, optimal heating of cystic masses and less procedural pain [3, 10, 33].
- *LITT* fibres are fully compatible with magnetic resonance imaging (MRI) and may be used in the presence of metallic implants. As the ablation zone size is limited (to 16 mm in diameter for 1200 J), its use is typically confined to the treatment of small (≤ 1 cm) benign tumours such as OO [34].
- *HIFU* is indicated for palliation of painful osteolytic, osteoblastic or mixed bone metastases in the event of radiotherapy failure or in cases where radiotherapy is contraindicated or declined by the patient. In select cases, where expected survival exceeds 1 year, or in the case of oligometastatic disease, local control may be pursued [26, 30]. Benign bone tumours may also be treated, most notably OO; however, HIFU is not recommended where cortical thickening exceeds 6 mm [35].
- The major advantages of CA are the precise visual control of the aggregated ice ball under both CT and MRI and much-reduced peri- and immediate post-procedural pain due to a relative anaesthetic effect. The combination of precise visual control and the ability to cover a large volume of tissue with multiple cryoprobes and overlapping ice balls ‘shaped’ to the morphology of the lesion while causing less pain than the heat-based modalities indicate cryoablation in the treatment of very large lesions (> 4 cm) with complex morphology and in tumours close to at-risk organs. Tumours close to metallic implants may be safely treated without the potential risks of electrical conductivity and adverse thermal effects associated with RFA and MWA.
- For the heat-based modalities, large lesions may be treated with multiple applicators producing overlapping ablation zones [33]; however, as above, control is optimal with cryoablation.

## Patient Preparation

### Pre-Procedural Preparation

#### Common to All Modalities

*Pre-procedural imaging* is directed at choosing the optimal approach to the lesion and identification of at-risk structures to plan the deployment of the probe and tissue monitoring/protection devices. Planning studies should be no more than one month old; re-imaging closer to the procedure may be necessary in lesions with aggressive tumour biology or if a patient’s symptoms have changed since the most recent imaging.

For osseus detail, non-contrast, thin section (1–3 mm) *computed tomography (CT)* with reconstructions in multiple planes is the recommended pre-treatment imaging for all osseus lesions [35–37]. Specifically with respect to OO, MRI is limited by lower sensitivity compared to CT in the clear demonstration of the nidus, as the latter can be isointense to cortical bone. Higher sensitivity and specificity rates are achieved with MRI using dynamic contrast enhancement and improved spatial resolution techniques [36, 38, 39]. Technetium-based radionuclide studies will show intense nidus uptake [36]. Contrast-enhanced CT and MRI followed by percutaneous biopsy may be necessary for the pre-treatment evaluation of other benign osseous tumours.

*Multiparametric MRI* (including T1- and T2-weighted sequences with and without fat saturation, diffusion-weighted imaging and dynamic contrast-enhanced multiplanar T1-weighted sequences) is recommended for the evaluation of malignant lesions [40]. Apparent diffusion coefficient (ADC) and dynamic contrast-enhanced sequences provide useful functional information [37].

*Positron emission tomography-CT (PET-CT)* will confirm or refute disease as oligometastatic and demonstrates prognostic metabolic parameters, e.g. standardised uptake value and metabolic tumour volume [41]. When PET-CT is performed, bone scans may be considered optional, although technetium-based studies remain of higher sensitivity for osseus metastases [40, 41].

*Treatment planning for MR-guided HIFU (MRgHIFU)* involves a number of steps: calibration, loading, segmentation, planning and verification, the details of which are beyond the scope of this document; please see the excellent descriptions by Napoli [26].

### Anaesthetic Support

All the heat-based modalities are painful, and although some ablations may be performed under conscious sedo-analgesia [26, 35, 42], anaesthetic support is invaluable, including general anaesthesia (GA), for the treatment of exquisitely painful targets such as the nidus of OO, and in the treatment of small target lesions where patient movement could jeopardise the ablation or increase the risk to adjacent at-risk structures [35]. The presence of an anaesthetist increases technical and clinical efficacy and reduces complication rates. An immobile target is especially crucial for the remote targeting required for MRgHIFU; thus, in the treatment of OO in children, general anaesthetic is indicated. Similarly, general anaesthesia is indicated for MRgHIFU of upper trunk lesions [26] and for all ablations in children younger than 14 [35].

*Pre-procedural anaesthetic review* is recommended to assess the patient’s performance status and suitability for general anaesthetic, and to evaluate the feasibility of other techniques such as spinal anaesthesia (spinal, pelvic or proximal lower limb lesions) or regional nerve blocks in the peripheries [3]. The latter blocks may also be used to achieve prolonged post-procedural pain relief.

The final choice will depend on patient factors such as age, cardiovascular and respiratory status, renal function, and on lesion factors such as location, size and expected duration of procedure [26, 42].

Unlike the heat-based modalities, cryoablation has a relative anaesthetic effect and is thus associated with less procedural and post-procedural pain, permitting the performance of many ablations under conscious sedo-analgesia, or indeed local anaesthetic alone.

### Pre-Procedural Consultation

Pre-procedural consultation in the interventional radiology clinic (IROC) should include consent, including agreement with the patient of the clinical objective and physical examination particularly a neurologic evaluation, to serve as a baseline in the event of a procedure-related thermal nerve injury. In the week beforehand, work-up should include full blood count, coagulation screen, assessment of renal function and blood typing. Local/systemic infection should be excluded.

## Treatment

### Probe-Based Technologies (Common to All)

#### Imaging

Imaging is used for immediate pre-procedure planning, targeting and intra-procedural guidance. Fluoroscopy with the addition of cone-beam CT technology, CT (with or without CT-fluoroscopy) or MRI can be used for guidance either alone or in combination with 3D navigation or image-fusion systems. Where available, high-resolution multimodality image guidance with CT and fluoroscopy is particularly useful (e.g. in the treatment of spinal lesions) allowing quick and precise probe placement and the deployment of protective/monitoring devices, resulting in lower complication rates and facilitating the combination of additional procedures such as sequential application of embolisation, ablation and percutaneous screw fixation or cementoplasty when indicated [2, 3, 25].

A small proportion of lytic tumours may be targeted under US guidance alone if they are relatively superficial and where adjacent sensitive structures can be clearly seen and avoided. Specifically for HIFU, MR guidance (MRgHIFU) is preferred to ultrasound guidance in the treatment of bone lesions for its precise targeting, and when used, an immediate pre-treatment MRI should be performed in the expected treatment position, to refine the treatment approach [26].

#### Immediate Pre-Procedural Preparation

Ideally patients should be provided with patient-controlled analgesia (PCA), particularly if being treated under conscious sedation. For long procedures and lesions in challenging locations, a urinary catheter is recommended. The patient is positioned to optimise the approach to the lesion, achieving the shortest straightest possible path while avoiding non-target sensitive structures. Antibiotic prophylaxis is recommended when cement injection and/or screw placement is planned in combination with the ablation; otherwise, prophylaxis is not routinely indicated but may be used depending on operator and institutional protocols. Vital signs should be monitored throughout.

#### Procedure

The CIRSE checklist should be completed [43]. After local asepsis and anaesthesia to skin, subcutaneous tissues and periosteum (including under GA, so-called pre-emptive anaesthesia, shown to reduce post-operative pain [44]), an 11- or 13-gauge bone trocar is inserted to the lesion of interest under image guidance and satisfactory position confirmed. The ablation probe is introduced through the coaxial trocar which is then withdrawn to a point outside the expected ablation zone (~ 1 cm) to prevent contact between the active tip and the trocar [5, 32] so as to avoid a skin/subcutaneous fat burn arising from heat transmission via the trocar. Once satisfied with the relative positions of the probe and trocar, the ablation may proceed. At completion, 5–10 ml of ropivacaine 2 mg/ml are injected to the periosteum to reduce post-procedural pain [32]. Imaging is repeated at completion to evaluate for immediate complications.

In the spine, small tumours located in the ipsilateral half of the vertebral body may be entirely ablated with a unipedicular approach, whereas for tumours extending beyond the sagittal midline of the vertebral body, ablation with a bipedicular approach is recommended to achieve adequate margins.

### Modality-Specific Recommendations, Including Device Selection

*RFA* probes are metallic needles with exposed cutting (active) tips of varying and adjustable length with an insulated shaft. Straight probes are used for small discrete targets such as the nidus of an OO and expandable/multi-clustered (umbrella-configuration) devices are used in the treatment of lesions where a larger ablation volume is required. RFA probes may be monopolar (requiring grounding pads) or bipolar (grounding pads unnecessary).

Specifically for spinal metastases, bipolar radiofrequency electrodes (articulating or straight) are available with thermocouples (incorporated or separate) that can easily combine tissue protection, ablation and vertebral augmentation in a single treatment.

*MWA* is performed with a water-cooled interstitial antenna. No grounding pads are required and there is no interference with implanted electrical devices such as pacemakers. MWA energy profiles are based on the manufacturer’s recommended combination of power level (i.e. wattage) and exposure time (i.e. minutes) needed to obtain the desired size of the ablation area. For larger lesions, MWA can be performed with multiple applicators [33] to achieve overlapping ablation zones. For *superficial OO*, it is not necessary to penetrate the cortical bone as, if the applicator tip is positioned directly over the lesion, the intense heat will penetrate the cortex and effect ablation. In cases of cortical interruption over or adjacent to the lesion, the probe can be positioned directly into the lesion; however, in most cases, and particularly in osteoblastic lesions, probe delivery is coaxial via a large-bore trocar (11–13G) [32].

#### LITT

Infrared lasers are most commonly used [45], the fibre inserted coaxially under CT guidance (1 mm collimation) and the introducer withdrawn. For subperiosteal tumours, an 18-gauge spinal needle is used for coaxial delivery. For ablation, 2 W power is applied for 6–10 min depending on tumour size, producing a spherical volume of ablation 1.6 cm in diameter in bone. Unless the position of the fibre tip is changed, delivering more than 1200 J does not increase the volume of coagulation. Use of higher power (up to 60 W) results in charring, vaporisation and cavitation around the fibre tip which limits heat transmission and consequently ablation. These effects are reduced by the use of internally cooled lasers facilitating an increased ablation volume [46]. Adjacent metalwork is not a contraindication. The devices are MR-compatible and do not interact with pacemakers.

#### Cryoablation

17-gauge gas-driven cryoprobes are introduced via an 11 or 13G trocar under CT or MR guidance. Superficial lesions may be ablated without penetrating the cortex by placing the probe immediately adjacent to the target and ensuring that the ice ball covers the lesion. Different types of cryoprobes are available and can be activated simultaneously, resulting in different volumes and shapes of ice ball to conform to the morphology of the target lesion. When using multiple probes, great care is required to avoid penetration of a cryoprobe when introducing another, as puncture may result in catastrophic intracorporeal gas release. Growth of the ice ball is monitored using intermittent reconstructions in multiple planes to ensure adequate coverage of the tumour and a safety margin with vulnerable tissues.

*For curative cryoablation*, the margins of the ice ball should extend 5–8 mm beyond the tumour margins, given the reduced freezing efficacy at the margin of the ice ball.

Repeated freeze–thaw–freeze–thaw cycles are required (at least 2, encompassing a 10-min freeze, 9-min passive thaw and a further 10-min freeze) to increase cellular necrosis between − 20 and − 40 °C [47]. Following a complete ablation cycle, brief active thawing (using electrical methods rather than helium gas as used in older probes) is used to free the probe which is invariably stuck to the tissue. The probe may also be heated on withdrawal to effect tract-cautery if required.

#### MRgHIFU

Patient positioning is optimised to achieve the shortest beam path and a normal angle of incidence. The target lesion should be a minimum of 1 cm in depth from the skin surface, which should be free of hair. Scar tissue, hollow viscera, metallic foreign bodies and non-target bone should be avoided [26]. The transducer is coupled to the skin with a gel pad (moistened with degassed water) [35, 42].

Where cortical bone is intact over the lesion, the focal spot is positioned deep to the cortex. If there is a cortical breach at the lesion, the focus of the ultrasound beam is targeted to the lesion itself and immediate surrounding cortex [26].

In OO, the nidus is precisely targeted (to within 0.2–5.0 mm^3^) with a 5 mm margin to prevent undertreatment [35]. Full energy sonications with energies of 1500–3000 J are generally required to treat the periosteum and higher (2500–6000 J) needed to treat lesions where there is a cortical breach.

Individual sonications should be limited to a few seconds, reducing heat-sink effects and allowing more accurate temperature assessment. Multiple sequential sonications are required to achieve homogenous ablation. Sequences are performed between higher-energy treatment sonications to generate a real-time thermometric map, accurate to within approx. 1 °C, 1 mm spatial resolution and 3 s temporal resolution [30, 37]. The temperature of the soft tissue adjacent to the osseus target is measured, as direct temperature measurement within bone is limited [37]. Treatment target temperatures are: 65 °C for metastases [37] and 60 °C for OO [35]. Negligible tissue effects are anticipated outside the focal zone [48].

### Contraindications/Disadvantages/Limitations of Thermal Ablation

*Absolute contraindications* for these ablation techniques are rare but include lack of a safe access, acute immunosuppression, local or systemic infection, uncorrected coagulopathy and patient refusal to consent. *Relative contraindications* include very large lesions and proximity to a sensitive structure that cannot be monitored or protected. In the spine, unstable fractures and metastatic epidural spinal cord compression are the most frequent relative contraindications. In the treatment of lesions in weight-bearing bones*,* ablation should be used in combination with bone consolidation/augmentation for support and stability due to an increased risk of post-ablation fracture [2, 3, 10, 23, 25, 40]. In this setting, if stabilisation is not possible, ablation should only proceed with caution and consent.

- *All thermal modalities* are potentially limited in their ablation efficacy by heat- or cool-‘sink’ effects whereby the tissue temperature at the ablation site is cooled or warmed by nearby high-flow vascular structures.
- *RFA, MWA and LITT* are limited by their inability to visualise the ablation zone under CT or US guidance, resulting in difficulty achieving a precise area of necrosis and hindering the ability to guarantee the safety of temperature-sensitive structures.
- Specific disadvantages of *RFA* include sensitivity to heat-sink effect and poor efficacy in osteoblastic lesions [31]. Although not absolutely contraindicated in the presence of metallic surgical fixation devices, great care is required in the placement of the probes in their vicinity to avoid potential adverse heating and electrical effects. Bipolar RF probes are recommended in this setting as is the use of local tissue temperature monitoring and protection.
- Although larger ablation volumes can be achieved using multiple bare *laser* fibres arrayed at 1.5- to 2-cm spacing, other modalities are preferred, given the ease with which larger volumes may be treated.
- *HIFU* should be avoided in rib lesions where there is inadequate bone to prevent ablation of underlying lung parenchyma and should not be used for lesions in the skull or vertebral bodies. Although there are sporadic reports of its use to treat lesions in the posterior elements of the lower lumbar spine below the level of the conus medullaris [30, 48, 49] its use cannot be recommended currently in this location on the basis of the available evidence. HIFU should not be performed in a peri-articular location or within 10 mm of a major neural structure [30]. The need for surgical stabilisation due to risk of fracture is a contraindication, as is prior fixation [30]. MRgHIFU is contraindicated in patients in whom MRI and/or gadolinium is contraindicated [26].
- *Cryoablation* Although ice ball visualisation is somewhat limited in sclerotic lesions, it remains effective in this setting. Cryoablation may be safely used in the presence of metallic fixation devices in contact with the tumour.

### Tissue Monitoring and Protection

The first step in tissue protection is to carefully plan the trajectory of the ablation probe(s) (and MRgHIFU beam) so as to avoid injury to at-risk structures during probe delivery. Neural injury occurs at temperatures higher than 45 °C or below 10 °C. Temperatures up to 42–45 °C or down to 0–10 °C can be neurotoxic proportional to the duration of exposure. Thus, during thermal ablation of benign or malignant lesions close to neural structures, monitoring and protection are strongly recommended [6, 32, 50].

Techniques to avoid thermal injury to non-target sensitive structures may be classified as (i)* passive monitoring* or (ii)* active protection* [50–53].

*Passive monitoring* is best exemplified by the use of thermosensors for direct temperature measurement in the immediate vicinity of an at-risk structure. Non-invasive MR-thermometry is used during MR-guided HIFU. In the treatment of vertebral lesions, one or more thermocouples (or a fibre-optic thermosensor when using MR guidance) are positioned in the epidural space via sublaminar or transforaminal approaches to constantly monitor the temperature in the neural foramina and spinal canal; ablation should be ceased when a temperature of 45 °C is reached [50].

For procedures performed under GA, peripheral nerve function may be monitored using electrophysiologic monitoring [somatosensory and motor-evoked potentials (MEP)]. Transcranial electrical impulses are recorded by percutaneous electrodes positioned over the muscles innervated by the nerves. When MEP decreases, ablation should be stopped immediately to prevent permanent nerve injury [50]. This intra-procedural monitoring also predicts motor deficits. Similarly, under GA, electrostimulators may be used in the periphery in contact with the major nerve, and evidence of reduced responsivity during the ablation should prompt cessation. Under general anaesthetic, muscle paralysis via neuromuscular blockade must be avoided, as it compromises electrophysiologic monitoring (via evoked potentials or electrostimulation).

If performing thermal ablation under conscious sedation (heat or cold-mediated), clinical monitoring via direct patient interaction, e.g. asking the patient to move intermittently or report altered sensation, is used to detect neural injury.

*Active protection* techniques include active *temperature modulation* adjacent to the ablation zone, and *displacement* techniques. These effects can be combined, e.g. hydrodisplacement using room-temperature fluid. Non-ionic dextrose 5% is used during RFA; saline or dextrose may be used during cryoablation. In the epidural space, combined hydrodissection and thermal monitoring are recommended. Continuous irrigation with epidural or periradicular infusion of normal saline is recommended when the target is less than 8 mm from adjacent nerve roots [34] as displacement effects are limited in these settings.

Non-neural structures, e.g. ureter and bowel, may be protected via active thermocouple temperature monitoring during RFA or MWA. Structures can also be insulated or displaced by carbon dioxide (carbon-dissection) or angioplasty balloons [50]. Hydrodisplacement with 0.9% saline can insulate the pericardium when treating sternal lesions [54].

For lesions close to skin, e.g. superficial anterior tibial cortical OO, physical displacement may be effected by subcutaneous injection of local anaesthetic or dextrose 5% injection. Warmed saline may also be injected subcutaneously to increase the ice ball-skin distance. Gas dissection and continuous temperature measurement in the subcutaneous space can also increase safety. Sterile gloves containing warm or cooled normal saline applied to the overlying skin provide protection against cold and heat, respectively [32].

Adjacent tissues cannot always be protected. While the ablation of lesions close to osteochondral structures (such as the periacetabular region) should be avoided where possible due to the risk of chondrolysis and femoral head osteonecrosis [21, 50], the presence of untreated tumour will result in residual/recurrent disease, and thus, the risk to the cartilage may have to be accepted to achieve tumour control. The patient should be warned and consented for this possibility. Similarly, cryoneurolysis may be acceptable in achieving adequate palliation in severe cases, if consent has been obtained.

### Combining Thermal Ablation with Other Required Treatments

#### Consolidation

In load-bearing (vertebrae, femoral head and neck, and acetabulum) and long bones, ablation and consolidation can be usefully combined to effect ablation and palliation and prevent or stabilise pathological fractures [2–4, 55–57]. If the goal of the treatment is curative, percutaneous osteoplasty should always be preceded by a definitive ablative treatment [3]. Thermal ablation can be combined in a single session with osseous consolidation using cement plus/minus augmentation with metallic fixation/stabilisation devices [3, 10].

For probe-based technologies, polymethylmethacrylate may be injected following ablation through the same trocar used to introduce the ablation device [3, 10]:

- If performed in the same session as one of the heat-based modalities, cement injection should be delayed to allow the local temperature to reduce in order to prevent too-rapid cement consolidation.
- Following CA of spinal or acetabular tumours, cement osteoplasty should be performed in combination with cryoablation to avoid a compression fracture. The cement is injected after complete thawing of the ice ball (tissue temperature should exceed + 20 °C) or delayed until the following day.

#### Embolisation

Embolisation may be used on its own in the palliation of hypervascular painful bone metastases, tumour ischaemia resulting in reduced intra-tumoural turgor and periosteal decompression leading to pain relief. Embolisation can be contributory when performed prior to thermal ablation in improving local tumour control (by limiting heat- or cool-sink effects due to adjacent high-flow vessels) and reducing haemorrhage during ablation and/or adjunctive osteoplasty/osteosynthesis [2]. Embolisation and consolidation may proceed in circumstances where heat-based ablation techniques are contraindicated.

#### Timing of Ablation with Respect to These Other Required Treatments and Radiotherapy

Where both vertebroplasty and radiotherapy are indicated, their relative timings are a matter of debate in the literature; however, some recommendations are possible based on the nature of the lesion and the radiotherapy modality utilised.

- In cases of solitary or oligometastases, it is recommended to perform thermoablation first, to effect local tumour control, followed by vertebroplasty to effect vertebral stabilisation and pain control. In radiosensitive lesions, subsequent radiation therapy can consolidate this treatment and improve the tumour response.
- Where the risk of pathological fracture is high, e.g. in predominantly lytic lesions, particularly those with massive osteolysis, vertebroplasty should be performed first to diminish the risks of pathological fracture/collapse and secondary neurologic compromise, followed by radiotherapy to effect local tumour control.
- Where a vertebral lesion is causing epiduritis, particularly with neurologic symptoms, radiotherapy should be performed first, followed by vertebroplasty.
- Where SBRT is planned with curative intent, some centres prefer that this is performed before vertebroplasty, given the potential risk of cement displacing tumour cells into the circulation.
- Where SBRT is used to treat small vertebral metastases to effect local tumour control and pain management, subsequent vertebroplasty is recommended to reduce the risk of collapse due to SBRT-related osteonecrosis.
- If transarterial embolisation and RT are both indicated, embolisation should follow RT as the efficacy of the latter is reduced by hypoxia [58].

Despite these recommendations, it is acknowledged that pragmatic factors such as local expertise and availability of each treatment modality will play a role in decision making and may dictate the actual timings in practice.

### Immediate Post-Procedural Imaging

Immediate post-procedural imaging using the guidance modality is directed at identifying immediate/early post-procedural complications, e.g. CT to evaluate for haemorrhage, fracture or cement leak [3]. Non-perfusion and increased ADC values in the target confirm ablation [26], deemed complete when the non-perfused volume encompasses the defined lesion and required margin. In palliation, the non-perfused volume should include the periosteum.

### Clinical Evaluation Immediately Post-Procedure

The skin should be examined for evidence of thermal injury. Vital signs should be observed for a minimum of 2 h. The requirement for subsequent monitoring will depend on the anaesthetic approach.

## Medication and Peri-Procedural Care

Post-procedural pain should be anticipated and is typically maximal in the first 24 h; however, the incidence is significantly higher and the intensity of pain experienced greater following heat-based treatments. Systemic analgesic medications, e.g. parenteral paracetamol, should be prescribed as a routine in addition to anti-inflammatories and should be administered prior, during and post-intervention. Following MRgHIFU ablation of OO in children, 3 doses of betamethasone (4 mg every 12 h) are administered to reduce post-ablation inflammation [35]. Patient-controlled analgesia (PCA) containing an infusion of opioid analgesia and anti-emetic medication is beneficial post-procedure. Post-procedural pain is less commonly reported following cryoablation due to its relative anaesthetic effect.

### Discharge

Patients are usually kept in hospital overnight to ensure adequate recovery from anaesthesia and optimal pain management, especially true for children [26]. The majority of patients will be discharged on the first post-operative day. Even when combining ablation with augmentation techniques in weight-bearing locations, early mobilisation is encouraged; however, advice should be given to avoid excessive stressful weight-bearing and strenuous activity for 2–3 weeks.

## Post-Procedural and Follow-Up Care

### Post-Treatment Follow-Up Care and Imaging Directly Related to the Treatment

Early follow-up consists of clinical evaluation immediately, 4 h, 1 day and 1 week post-ablation in order to exclude early complications and to assess the adequacy of the treatment with respect to pain relief.

Delayed follow-up is performed regularly in the months to years following ablation and consists of a thorough interventional radiology outpatient clinic (IROC) assessment as well as biological testing, including tumour markers to ensure clinico-biological stability [59, 60].

Imaging is targeted at (a)* the ablated lesion* to assess response and exclude residual or recurrent disease, or complications such as infection and (b)* staging of the underlying pathology*, including the development of progressive disease elsewhere or metachronous oligometastases.

In the curative setting, MRI and PET-CT are the preferred techniques to detect local residual tumour or early relapse [4, 38, 39, 61]. In the palliative setting, clinical follow-up suffices, unless complications are suspected. Interval imaging may be triggered at any point if a complication or recurrence is suspected. Suspicion of fracture should be evaluated with radiographs and CT. Osseus remineralisation on CT is considered an indicator of successful ablation [26]. MRI and CT are typically performed at 1, 3, 6 and 12 months following MRgHIFU.

Paediatric patients who have undergone MRgHIFU for OO are followed with unenhanced MRI at 6, 12 and 24 months looking for changes in marrow oedema and inflammation in the soft tissues adjacent to the lesion [35].

### Clinical Assessment of Outcome

When performed for pain relief, post-procedural pain should be assessed using a validated tool such as a visual analogue score. Analgesic requirements should be documented and the brief pain inventory quality-of-life score measured at 1, 3, 7 and 14 days post-treatment, and at 30-day intervals for one year following treatment [26]. In children, the response should be assessed using the Faces Pain Score—Revised, analgesic requirements ± the Paediatric Initiative on Methods, Management and Pain Assessment in Clinical Trials (PEDIMMPACT) [35, 62, 63].

### Complications

Clinically significant complications are not common (approx. 2.3%) following *RFA* of bone lesions, fracture being the most common (1.8%). Other rarely encountered complications include infection, skin burn, peripheral sensory or motor neuropathy, arthropathy and haematoma [51]. Factors associated with a higher complication rate are a tumour size > 3 cm and previous radiotherapy, the latter the only risk factor specifically associated with an increased minor complication rate [51]. Although rare, neural thermal injury is one of the more frequent complications following RFA. The majority of patients will recover completely from such unintended ablation; however, they must be appropriately advised regarding the likely duration of recovery (between 6 and 18 months).

Both *RFA* and *MWA* can cause iatrogenic skin burns [5, 32] if a non-insulated applicator or trocar is used. A systematic review of *MWA* revealed clinically significant complications, including skin burns in 10/249 (4.0%) patients [64].

Reflex sympathetic dystrophy resulting in persistent pain for 2 months has been reported rarely following *LITT* [34]. Its occurrence can be avoided by insulation techniques; to further reduce this risk, regional block prior to ablation of extremity nidi is recommended.

*Cryoablation* is a safe treatment of bone tumours with a very low rate of major complications (2.5%), the most common of which is secondary fracture (1.2%). Risk factors associated with major complications are: patients older than 70 and the use of more than three cryoprobes. Minor complications occur more often in patients with a poor performance status (Eastern Cooperative Oncology Group (ECOG) performance status > 2), ablation of lesions in long bones and the use of more than 3 cryoprobes [65].

Temporary neurolysis is reported in up to 6% of cases where nerves are exposed to temperatures less than 10 °C [66]. Freezing of a peripheral nerve produces a predictable lesion with prolonged interruption of conduction; however, this lesion is usually reversible and normal morphology of the nerve often reappears [67]. If a nerve is included in the centre of the ice ball, where temperatures of − 40 °C or lower predominate, permanent neurological damage should be expected [68]. Very infrequently observed complications are: site infection, tumour seeding, bleeding and severe hypotension (0.3%) [65].

Other sporadic complications reported following cryoablation include haemothorax and avascular necrosis leading to femoral head collapse requiring hip replacement 8 months after cryoablation of a periacetabular chondrosarcoma metastasis [21]. Cryoshock [69, 70] has not been reported in osseus cryoablation.

Following *HIFU*, possible minor treatment-related side effects include sonication pain, early post-procedural pain or bruising in the treated area, position pain, first- and second-degree burns less than 2 cm in diameter and transient fever. These generally resolve within 2 weeks of treatment with no long-term sequelae. Possible major side effects include necrosis of non-target tissue, perforation of hollow viscera, third-degree skin burns with ulceration, fracture hip flexor neuropathy and anaesthetic complications [26].

## Outcomes

Thermal ablation of appropriately selected osseus lesions of varied histologies is associated with high success and low recurrence and complication rates, summarised in Table 1.

**Table 1:** Outcomes following thermal ablation of osseus lesions

	RFA	MWA	LITT	Cryoablation	MRgHIFU
Osteoid osteoma	Success rates: 91.7–96.9%	Success rates similar to RFA [32]	Success rates similar to RFA [34, 72–74]	Good pain reduction, with low rates of minor complications (14.3%) and recurrence (4.8%) [75]	97% complete resolution of pain, evidence of bone healing and no complications up to 24 months [35]
	No correlation between treatment failure and ablation duration, patient age or lesion location [12–14, 71]	Effective pain relief at 1 month in 92.3–100 [64]			
		In appendicular OO, skin protection is key			
Malignant lesions: curative intent	67% tumour-free survival 1 year post-RFA or CA in oligometastases [76]	No large series analysing the feasibility, safety and efficacy of MWA in the curative setting		In oligometastatic disease, local control in 68%, with 1- and 2-year survival of 91% and 84%, median disease-free survival of 7 months and disease-free survival of 25% and 7% at 1 and 2 years	
	100% overall survival at 1 year for sarcoma oligometastases [6]			Tumour-free survival of 21 months [21, 76]	
Malignant lesions: palliative intent	Particularly useful in patients with recalcitrant pain [77, 78]	Effective pain palliation in metastases [1, 4, 5, 23, 25]		Safe and effective method for pain palliation with early reduction in pain scores and analgesic requirements sustained at 1 month, with further improvement in the following 6 months [79–81]	Pain relief in 2/3 patients with painful metastases within 3 days. 64.3% of patients improved pain score of at least 2 points on the numerical rating scale at 3 months
	Pain relief superior in axial compared to appendicular tumours	Estimated pain reduction was 5.3/10 at 1 month; and 5.3/10 at last review (20–24 weeks in 4/5 studies) [64]		Good local tumour control (79% at 12 months) [82]	23.2% of patients had a complete response [48]
	*Bipolar radiofrequency ablation* (b-RFA) with increased target temperature (> 70 °C) in combination with vertebroplasty achieved pain relief (80% efficacy) and local tumour control in oligometastatic/oligoprogressive lesions (100% efficacy) [78]				

### Benign Lesions

Success rates are uniformly high for all modalities treating OO. Experience with other benign bone tumours, e.g. ABC, osteoblastoma, is limited [15–20, 83].

### Malignant Bone Lesions

Good tumour-free survival rates (67–68% at one year) are reported for RFA, MWA and CA in oligometastatic metastases, including sarcoma [6, 21, 76]. Thermal ablation achieves effective pain relief [4, 5, 25, 48, 64, 66, 77–82, 84]. Multiple studies have found improved pain relief in spinal lesions when ablation was combined with vertebroplasty and/or RT [85–87]. In general, tumours less than 2 cm with no cortical erosion fare better [76]. The combination of cryoablation and bisphonates has been found to be synergistic in the setting of painful osseus metastases [88].

## Conclusion

Percutaneous thermal ablation has an established role in the successful management of a variety of benign and malignant bone lesions, with both curative and palliative intent. The selection of the most appropriate modality for a given target lesion in an individual patient should be made in a multidisciplinary team setting, where further decisions regarding tissue protection and adjunctive interventions such as cement osteoplasty, osteosynthesis and embolisation may also be made.

## Appendix 1: Key recommendations

- Each patient’s overall status and target lesion(s) should be discussed in a multidisciplinary team setting to assess the suitability and feasibility of a thermal ablative approach, to select the most appropriate technique and to set the goals of treatment, i.e. curative or palliative.
- Imaging studies used to plan the procedure should be no more than one month old.
- Antibiotic prophylaxis is recommended if combining cement osteoplasty and/or screw fixation with thermal ablation.
- Inclusion of periosteum in the ablation zone contributes significantly to pain relief in the palliative setting. Where an extra-osseus soft tissue component exists, ablation of the soft tissue–bone interface can achieve satisfactory palliation of pain.
- In the treatment of lesions in weight-bearing bones, ablation should be used in combination with bone consolidation/augmentation due to the risk of post-ablation fracture.
- During thermal ablation of lesions close to neural structures, passive monitoring and/or active protection is strongly recommended.
- If general anaesthetic is being used, muscle paralysis via neuromuscular blockade must be avoided, so as not to compromise electrophysiologic monitoring.
- If cement osteoplasty is performed in the same session as thermal ablation, the cement injection should be delayed so as to allow local temperature normalisation in order to ensure predictable setting of the cement.
- Where the risk of pathological fracture is high, e.g. in predominantly lytic lesions, particularly those with massive osteolysis, vertebroplasty should be performed first to diminish the risks of pathological fracture/collapse and secondary neurologic compromise, followed by radiotherapy to effect local tumour control.
- Where a vertebral lesion is causing epiduritis, particularly with neurologic symptoms, radiotherapy should be performed first, followed by vertebroplasty.
- Embolisation should be considered prior to thermal ablation to limit thermal-sink effects and/or to reduce bleeding during the ablation and/or adjunctive consolidation.
- If both are indicated, embolisation should follow RT as the efficacy of the latter is reduced by hypoxia.
- Regional anaesthetic block is recommended prior to ablation of extremity lesions to reduce the risk of post-procedural complex regional pain syndrome.
- Temporary neuropraxias may occur following thermal ablation, many of which will resolve; however, patients need to be warned that healing may take as long as 18 months.

## Abbreviations

ADC: Apparent diffusion coefficient
CA: Cryoablation
CT: Computed tomography
DWI: Diffusion-weighted imaging
ECOG: Eastern Cooperative Oncology Group
FBC: Full blood count
HIFU: High-intensity focused ultrasound
IROC: Interventional radiology outpatient clinic
LITT: Laser photocoagulation or laser interstitial thermotherapy
MDT: Multidisciplinary team
MEP: Motor-evoked potentials
MRgHIFU: MR-guided HIFU
MRI: Magnetic resonance imaging
MTV: Metabolic tumour volume
MWA: Microwave ablation
OO: Osteoid osteoma
PCA: Patient-controlled analgesia
PedIMMPACT: Pediatric Initiative on Methods, Management and Pain Assessment in Clinical Trials
PET-CT: Positron emission tomography-CT
PMMA: Polymethylmethacrylate
RFA: Radiofrequency ablation
RT: Radiation therapy
SBRT: Stereotactic beam radiation therapy
SOP: Standards of practice
SRE: Skeletal-related events
SUV: Standardised uptake value
US: Ultrasound
VAS: Visual analogue score
